# Acupuncture for Major Depressive Disorder: Exploring the Gut Microbiota as a Novel Therapeutic Pathway

**DOI:** 10.1002/brb3.71022

**Published:** 2025-10-29

**Authors:** Cailing Wei, Yijun Li, Jiarong Tian, Pu Lei, Yuanyuan Ding, Wen Lu, Xiaoyan He, Ya'ni Yang, Hao Zhu, Ruina Liu

**Affiliations:** ^1^ Department of Psychiatry The First Affiliated Hospital of Xi'an Jiaotong University Xi'an Shaanxi China; ^2^ Center for Translational Medicine The First Affiliated Hospital of Xi'an Jiaotong University Xi'an Shaanxi China; ^3^ Shaanxi Provincial Key Laboratory of Biological Psychiatry The First Affiliated Hospital of Xi'an Jiaotong University Xi'an Shaanxi China; ^4^ Department of Neurology the First People's Hospital of Xianyang Xianyang Shaanxi China; ^5^ Xi'an Mental Health Center Xi'an Shannxi China; ^6^ Department of Psychiatry the Ankang Central Hospital Ankang Shaanxi China

**Keywords:** acupuncture, association analysis of 16sRNA with non‐target metabolome, major depression disorder

## Abstract

**Aims:**

This study investigates the efficacy of acupuncture at Baihui (GV20) and Zusanli (ST36) acupoints in alleviating depressive symptoms and elucidates the underlying mechanisms.

**Methods:**

Sixty male C57BL/6J mice were subjected to an 8‐week chronic unpredictable mild stress (CUMS) paradigm. Acupuncture was administered to two groups during the final 3 weeks: one under CUMS and the other without stress. Behavioral assessments, including the sucrose preference test and tail suspension test (TST), evaluated depression‐like behaviors. 16S rRNA sequencing and non‐targeted metabolomics analyzed gut microbiota and metabolites, respectively, with association analysis exploring the mechanistic pathways.

**Results:**

Acupuncture significantly ameliorated CUMS‐induced depression‐like behaviors, restoring sucrose preference (from a significant reduction in CUMS, *p* < 0.001, to an increase in CUMS + AP, *p* = 0.035) and reducing immobility time in the TST (*p* = 0.045). 16S rRNA sequencing revealed that acupuncture partially restored the CUMS‐disrupted *Bacteroidetes*/*Firmicutes* ratio by specifically decreasing the pathobiont *Clostridium* sp. *A3LF 105b* and increasing beneficial bacteria such as *Faecalibacterium* and *Lachnospiraceae UCG 001*. These microbial shifts were functionally linked to the elevation of the critical metabolite sphinganine 1‐phosphate (S1P). Spearman correlation analysis revealed a negative correlation between *Clostridium* sp. *A3LF 105b* and S1P (*p* < 0.01, *R* < −0.52). The modulation of gut microbiota led to increased S1P, which in turn activated the “neuroactive ligand–receptor interaction” pathway, thereby restoring neuronal activity and alleviating depressive behavior.

**Conclusion:**

The study underscores the potential of acupuncture as an antidepressant treatment, highlighting its impact on gut microbiota and metabolic pathways in alleviating depressive symptoms.

## Graphical abstract

Acupuncture at GV20 and ST36 alleviates depression by modulating gut microbiota, increasing beneficial bacteria, and enhancing neuroactive metabolites like Sphinganine 1‐phosphate, thereby restoring neuronal activity and reducing depressive behaviors.

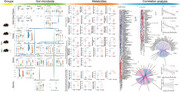

## 1 Introduction

Major depressive disorder (MDD), a debilitating psychiatric condition affecting over 300 million individuals globally, represents a critical public health challenge with substantial socioeconomic burdens (De Oliveira Rodrigues et al. 2023). Current pharmacological interventions exhibit limited efficacy and significant adverse effects in many patients, necessitating exploration of alternative therapeutic strategies (De Oliveira Rodrigues et al. 2023; Chang et al. 2023). Emerging evidence implicates gut microbiome dysregulation as a pivotal peripheral regulator of MDD pathogenesis, modulating neuroimmune signaling, neurotransmitter synthesis, and stress‐axis activity (Zhang, Shen, et al. 2022; Crost et al. 2023). Notably, microbial metabolites and neuroactive compounds influence key neurobiological substrates implicated in depression, including the hypothalamic–pituitary–adrenal (HPA) axis, neurotrophic factor regulation, and neurotransmission. Acupuncture, a cornerstone of traditional Chinese medicine, demonstrates therapeutic promise for MDD through neuromodulatory and anti‐inflammatory mechanisms (Liu et al. 2021; Yin et al. 2022). By stimulating specific acupoints (e.g., GV20 and ST36), acupuncture regulates physiological homeostasis via bidirectional gut–brain communication pathways. The Baihui (GV20) acupoint, situated at the vertex along the governing vessel meridian, exerts neuroprotective effects by mitigating excitotoxicity, promoting hippocampal neurogenesis, and enhancing synaptic plasticity (Xue et al. 2021; Zhang, Fan, et al. 2022; Wang, Liu, et al. 2021). Experimental models reveal GV20 stimulation attenuates anxiety‐like behaviors and modulates astrocyte–neuron interactions critical for cognitive function (Chen et al. 2023). Similarly, Zusanli (ST36), located on the stomach meridian, activates vagal–adrenal anti‐inflammatory pathways, elevates interleukin‐4 (IL‐4) and MyD88 signaling, and ameliorates inflammation‐associated depressive phenotypes (Yang et al. 2024; Chen et al. 2019; Qin et al. 2020).

Of particular translational relevance is acupuncture's ability to restore gut microbial equilibrium during neuropsychiatric disturbances (Zhang et al. 2019). Dysbiosis of gut microbiota, linked to impaired barrier integrity, systemic inflammation, and neurotransmitter imbalance, correlates with depressive symptom severity. Mechanistically, microbial‐derived metabolites (e.g., short‐chain fatty acids and tryptophan derivatives) regulate serotonin synthesis, microglial activation, and neuroinflammatory cascades (Liu et al. 2024; Jiang et al. 2024). Although preclinical studies associate acupuncture with compositional shifts in *Bacteroidetes*, *Firmicutes*, and *Lactobacillus* species, the precise microbiota‐mediated pathways underlying its antidepressant efficacy remain underexplored.

This study posits that electroacupuncture targeting GV20 and ST36 alleviates MDD pathophysiology by modulating gut microbiome structure and metabolic activity. We hypothesize that such interventions restore microbial diversity, suppress inflammation, and enhance neuroprotective signaling cascades, thereby bridging intestinal ecology with central nervous system (CNS) plasticity. By elucidating these mechanisms, this work aims to advance therapies for MDD while addressing critical gaps in microbiota‐acupuncture interplay.

## 2 Experimental Procedures

### 2.1 Experimental Animals

Sixty adult male C57BL/6J mice (8 weeks old; 18–22 g) were housed under specific pathogen‐free (SPF) conditions (21°C ± 2°C, 40% ± 5% humidity, and 12 h light/dark cycle) in groups of five per cage. Following a week acclimatization period, mice were randomly allocated into four experimental groups: (i) control (Ctrl), (ii) control with acupuncture (Ctrl + AP), (iii) chronic unpredictable mild stress (CUMS), and (iv) CUMS with acupuncture (CUMS + AP). Male mice were prioritized for neurobehavioral and gut dysbiosis analyses to minimize variability from estrous cycle–dependent hormonal fluctuations, which are known to influence stress‐related phenotypes and microbial community dynamics. All procedures strictly adhered to the ARRIVE guidelines and were approved by the Experimental Animal Ethics Committee of Xi'an Jiaotong University (No. 2022‐497). Efforts to minimize pain and discomfort included environmental enrichment and humane endpoint protocols.

### 2.2 CUMS Protocol and Acupuncture Intervention

The CUMS model was employed to simulate chronic low‐intensity stressors encountered in daily life (Hao et al. 2024). With the exception of control (Ctrl) and control plus acupuncture (Ctrl + AP) groups, all cohorts underwent a standardized CUMS protocol comprising the randomized stressors administered daily for 8 consecutive weeks. These included circadian disruption (24 h light/dark inversion), resource restriction (24 h food/water deprivation), physical stressors (2 h restraint, 5 min cold swim at 4°C), and environmental challenges (wet bedding, cage tilt, and strobe lighting).

Commencing at Week 5 of CUMS exposure, therapeutic acupuncture was administered to Ctrl + AP and CUMS + AP groups for 20 min/day over 3 weeks, consistent with established protocols (modified from standard methodologies). Baihui (GV20) acupoint was located in the middle of parietal bone. The Zusanli acupoint (ST36) was located on the posterolateral side of the knee joint, about 2 mm below the small head of the fibula, with bilateral acupuncture. Sterilized stainless steel needles (0.18 × 13 mm^2^) were inserted perpendicularly (2–3 mm depth) and rotated 180° bidirectionally every 5 min to enhance stimulation efficacy.

### 2.3 Behavioral Assessments

#### 2.3.1 Sucrose Preference Test (SPT)

Anhedonia‐like behavior was quantified via a standardized sucrose preference paradigm. During habituation (Day 1), mice were singly housed with dual access to 10 g/L sucrose solution for 24 h. One bottle was replaced with normal drinking water, whereas the other remained sucrose solution. Bottle positions were alternated every 12 h to mitigate positional bias at the raining phase (Day 2). On the third day, the sugar water preference test was carried out for 24 h after 12 h of no water and no fasting. During the testing phase, the positions of sugar water and normal water were changed after 12 h, and the sugar water consumption and total liquid consumption of the mice within 24 h were recorded: sucrose preference = sucrose solution consumption/(sucrose solution consumption + normal water consumption) × 100%.

#### 2.3.2 Tail Suspension Test (TST)

Depressive‐like immobility was assessed by suspending mice 30 cm above a platform using adhesive tape positioned 1 cm from the tail tip. After 1 min acclimatization, total immobility duration (passive hanging without escape attempts) during the subsequent 5 min interval was quantified using automated ANY‐maze software (v6.0, Stoelting Co., USA) and cross‐validated through blinded manual scoring.

### 2.4 rRNA Gene Sequencing

Fecal genomic DNA was extracted using QIAamp Fast DNA Stool Mini Kit (Qiagen) with quality verification through Qubit quantification and gel electrophoresis. The hypervariable V3–V4 regions of bacterial 16S rRNA genes were amplified using barcoded primers 338F/806R, followed by AMPure XP bead purification and Illumina‐compatible library preparation. Paired‐end sequencing was performed on DNBSEQ‐T7 (MGI Tech). Bioinformatic processing involved adapter trimming and quality filtering in Trimmomatic, read merging with FLASH (±10 bp overlap), and chimera removal via UCHIME. High‐quality sequences were clustered into 97% similarity OTUs using QIIME2, with taxonomic classification against SILVA 138.1. Microbial diversity analysis included α‐diversity metrics (Shannon, Chao1) computed in Mothur and β‐diversity assessment through Bray–Curtis‐based PCoA visualization in QIIME2.

### 2.5 Non‐Targeted Metabolomic Analysis

Untargeted metabolomics analysis was conducted using LC–MS data processed through an optimized bioinformatics pipeline. Raw spectra underwent mzXML conversion (MSConvert) followed by XCMS‐based feature extraction with retention time alignment and peak matching (Smith et al. 2006; Navarro‐Reig et al. 2015). Metabolite identification employed high‐resolution mass matching (<30 ppm) against multi‐repository spectral databases (HMDB, LipidMAPS, and Kyoto Encyclopedia of Genes and Genomes [KEGG])) with MS/MS spectral validation (Wishart et al. 2007; Horai et al. 2010; Sud et al. 2007; Abdelrazig et al. 2020; Ogata et al. 1999). Data normalization utilized QC‐RLSC with LOESS correction, retaining features exhibiting <30% relative standard deviation (RSD) in quality controls (Gagnebin et al. 2017). It effectively filters out metabolic features with high technical variability, thereby increasing the reliability of the biological conclusions drawn. Orthogonal partial least square‐discriminant analysis (OPLS‐DA) modeling (200 permutations) identified significant metabolites (VIP > 1.0, *p* < 0.05) (Xia and Wishart 2011). Pathway analysis in MetaboAnalyst 5.0 integrated hypergeometric testing and betweenness centrality metrics (false discovery rate [FDR] < 0.2), with KEGG Mapper visualization for biological interpretation of perturbed metabolic networks. This rigorous workflow ensured comprehensive metabolite profiling and mechanistic insight generation.

### 2.6 Statistical Analysis

Metagenomic abundance profiles and LC–MS/MS metabolomic data were subjected to rigorous quality control. Features exceeding 30% RSD in QC samples were excluded. Metabolomics data underwent total peak area normalization, followed by quantile normalization and log 2‐transformation to mitigate technical variability. This is to correct the differences in sample concentration and instrument analysis and make the data distribution more symmetrical, which is crucial for subsequent statistical analysis. Metagenomic taxonomic/pathway abundance data retained their compositional nature without transformation. Distribution properties were systematically evaluated using Shapiro–Wilk tests (*α* = 0.05). Metabolism data: 62% of metabolites violated normality assumptions (Kolmogorov–Smirnov *p* < 0.01). Microbiology data: >85% features showed right‐skewed distributions (Shapiro–Wilk *p* < 0.05). Metabolomics: Kruskal–Wallis test with Dunn's post hoc correction, supplemented by parametric OPLS‐DA modeling (VIP > 1, permutation‐validated R2/Q2 metrics). The nonparametric Mann–Whitney *U* tests were used to compare metabolite levels between groups, generating raw *p* values for each metabolite. The Benjamini and Hochberg correction was applied using R (specifically the p.adjust function with method = “BH”) (Wang et al. 2015). BH method can control the false discovery rate (FDR). It can not only identify statistically significant features but also effectively limit the Type I errors (false positives). This function automates the ranking and adjustment process described above. Adjusted *p* values (FDR‐corrected) <0.05 were considered statistically significant. Multi‐omics integration: Spearman's rank correlation (|*ρ*| > 0.5, FDR‐adjusted) for microbiota–metabolite relationships (Lu et al. 2025). The |*ρ*| > 0.5 threshold was used for an initial broad selection of associations. Importantly, our graphical and biological interpretation prioritized the strongest correlations with an FDR‐adjusted *p* value <0.05, ensuring the robustness of our key findings. Dimensionality reduction employed PCA, PLS‐DA, and OPLS‐DA (Ropls package) with visualization through ggplot2. Pathway enrichment analysis combined hypergeometric testing (KEGG Mapper) and topology impact scores (Mu et al. 2025). Analyses were conducted in R v4.3.2 using phyloseq (microbiome), MetaboAnalystR (metabolomics), and mixOmics (integration). Multiple testing correction uniformly applied Benjamini–Hochberg (microbiome) or *q* value (metabolism) methods, ensuring robustness to non‐normal distributions (Sun et al. 2024).

## 3 Results

### 3.1 Depression‐Like Behaviors Are Accompanied by Gut Dysbiosis

**FIGURE 1:**
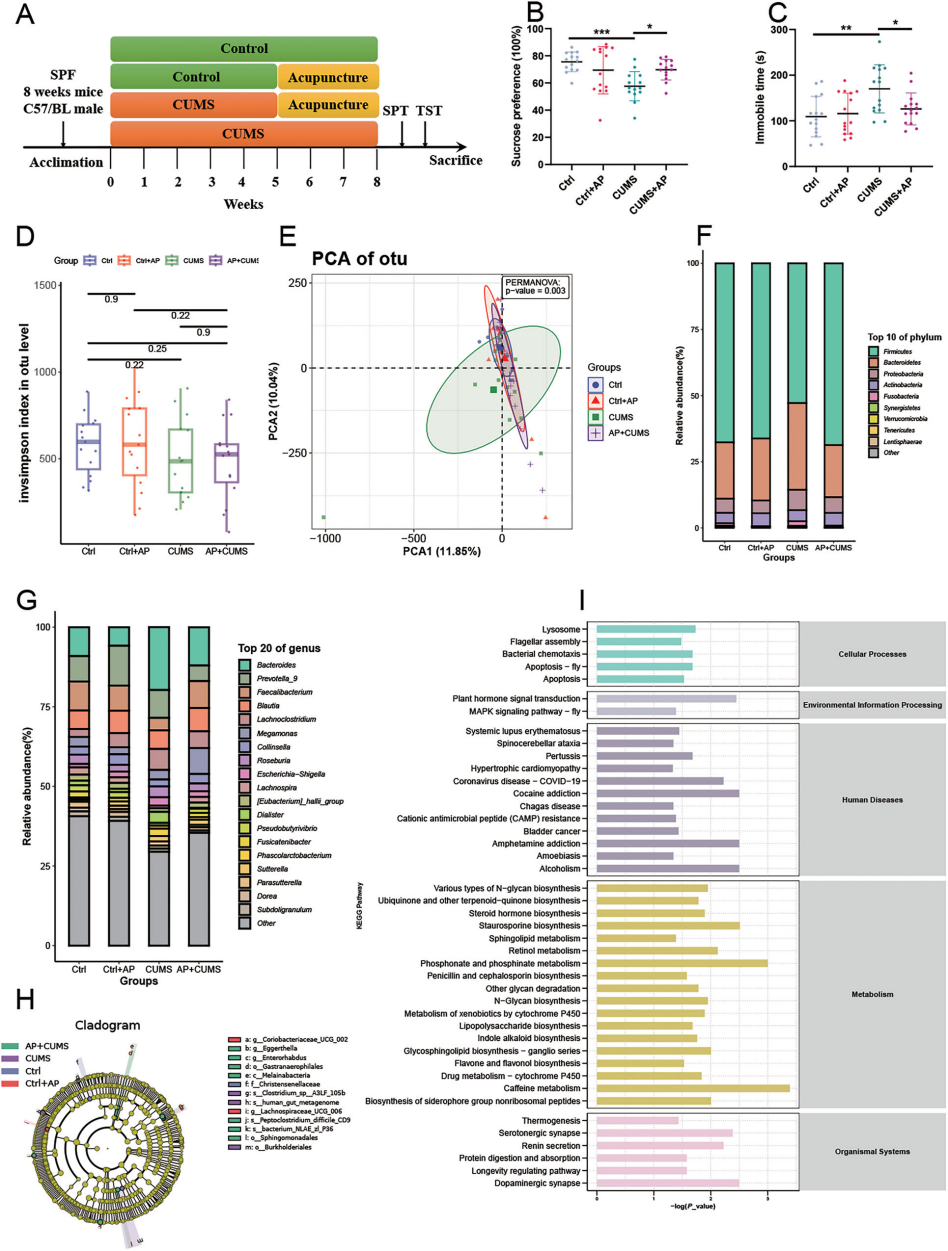
**Acupuncture attenuates CUMS‐induced depression‐like behaviors and modulates gut microbiota composition**. (A) Experimental schematic illustrating CUMS protocol implementation and acupuncture intervention. (B) Sucrose preference test revealed significantly reduced hedonic behavior in CUMS mice compared to controls (*p* < 0.001), with partial rescue in the acupuncture‐treated group (CUMS + AP vs. CUMS: *p* < 0.05). Data are presented as means ± SEM. (C) CUMS mice exhibited prolonged immobility time in tail suspension tests (*p* < 0.01 vs. control), attenuated by acupuncture intervention (*p* < 0.05). Statistical significance tested via one‐way ANOVA followed by Tukey's multiple comparison test. (D) The α‐diversity was assessed using Shannon indices at the OUT level in microbial communities. The Kruskal–Wallis rank sum test was used to analyze the differences. (E)The β‐diversity principal coordinate analysis (Bray–Curtis distance; PERMANOVA *p* = 0.002) revealed distinct microbial clustering patterns between groups. (F) Phylum‐level analysis identified *Bacteroidetes*/*Firmicutes* ratio alterations in CUMS mice (Wilcoxon rank‐sum test, *p* < 0.05). (G) Genus‐level comparison (Wilcoxon rank‐sum test, *p* < 0.05) highlighted 20 differentially abundant taxa between experimental groups. (H) The LDA score shows species with significant abundance differences in four groups and the length of the bar chart represents the impact of significantly different species (linear discriminant analysis effect size, LEfSe; (LDA) > 4). (I) The OUT sequences of the abundance differences between groups were compared with the Kyoto Encyclopedia of Genes and Genomes (KEGG) database using the PICRUSt software to obtain the intestinal microbiome gene functions with differential enrichment between control and CUMS groups. Ctrl + AP, the control + acupuncture mice; Ctrl, the control mice; CUMS + AP, the CUMS + acupuncture mice; CUMS, chronic and unpredictable mild stress. *p < 0.05, **p < 0.01, and ***p < 0.001.

To investigate depression–gut microbiota interactions, we established a CUMS mouse model followed by acupuncture intervention at Baihui (GV20) and Zusanli (ST36) acupoints over 8 and 3 weeks, respectively (Figure 1A). CUMS mice exhibited hallmark depressive phenotypes, demonstrating significantly reduced sucrose preference (*p* < 0.001) and prolonged immobility time in TSTs (*p* = 0.003) compared to controls (Figure 1B). Acupuncture notably mitigated these behaviors, restoring sucrose preference (*p* = 0.035) and reducing TST immobility (*p* = 0.045) (Figure 1C).

16S rRNA sequencing revealed gut microbiota restructuring despite preserved α‐diversity (Shannon index: *p* > 0.05; Figure 1D). β‐Diversity analysis via Bray–Curtis PCoA showed distinct clustering patterns (PERMANOVA *p* < 0.001 for control vs. CUMS; *p* = 0.031 for CUMS vs. CUMS + AP) (Figure 1E). At the phylum level, CUMS mice displayed reduced *Firmicutes* abundance with proportional increases in *Bacteroidetes* and *Proteobacteria* (Figure 1F). In terms of bacteria genus, the dominant genera that occupy the top 20 in the microbial community abundance of the four groups included *Bacteroides*, *Faecalibacterium*, and *Lachnoclostridium* (Figure 1G). There were significant differences in the abundance of intestinal flora in the four groups. In addition, the difference in the flora may contribute to the occurrence and development of the disease. A cladogram from LEfSe analysis of microbiome data revealed distinct abundant clades in each group (*p* < 0.05, linear discriminant analysis [LDA] > 5.0). *Christensenellaceae* is a beneficial characteristic marker of the control mice. *Clostridium* sp. *A3LF105b* was the characteristic markers of CUMS mice. *Gastranaerophilales*, *Enterorhabdus*, *Eggerthella*, and *Peptoclostridium difficile CD9* are increased in CUMS + AP mice (Figure 1H). In addition, the characteristic intestinal flora of each group can be used to distinguish the mice. The rough functional potential of the control and depressed microbial communities is based on the 16S rRNA gene content. Functional prediction highlighted enhanced neuroactive pathways in CUMS mice, including dopaminergic and serotonergic synapse regulation, alongside upregulated substance addiction and retinol metabolism pathways (*p* < 0.001) (Figure 1I). Acupuncture partially normalized these metabolic perturbations while activating phosphonate and caffeine metabolisms.

### 3.2 Acupuncture at GV20 and ST36 Acupoints Alleviated CUMS‐induced Depression‐Like Behaviors in a Gut Microbe‐Dependent Manner

**FIGURE 2:**
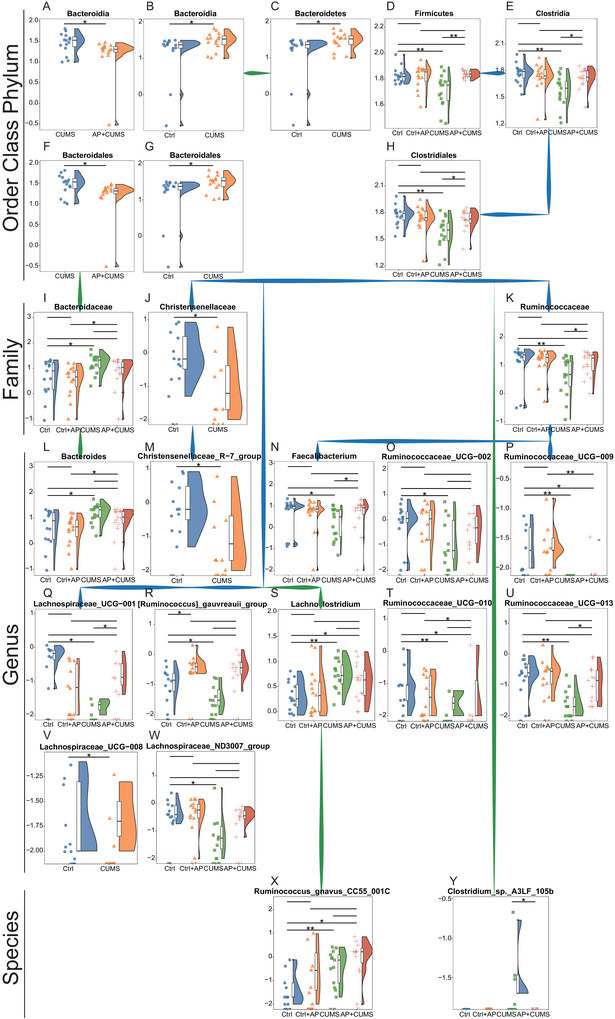
**Differential abundance of bacterial genera between control and depressive mice under CUMS**. (A) The differences in *Bacteroidia* between the CUMS group and the AP + CUMS group. (B) The differences in *Bacteroidia* between the control group and the CUMS group. (C) The differences in *Bacteroidetes* between the control group and the CUMS group. (D) The differences in *Firmicutes* among the four groups. (E) The differences in *Clostridia* among the four groups. (F) The differences in *Bacteroidales* between the CUMS group and the AP + CUMS group. (G) The differences in *Bacteroidales* between the control group and the CUMS group. (H) The differences in *Clostridiales* among the four groups. (I) The differences in *Bacteroidaceae* among the four groups. (J) The differences in *Christensenellaceae* between the control group and the CUMS group. (K) The differences in *Ruminococcaceae* among the four groups. (L) The differences in *Bacteroides* among the four groups. (M) The differences in *Christensenellaceae R‐7 group* between the control group and the CUMS group. (N) The differences in *Faecalibacterium* among the four groups. (O) The differences in *Ruminococcaceae UCG‐002* among the four groups. (P) The differences in *Ruminococcaceae UCG‐009* among the four groups. (Q) The differences in *Lachnospiraceae UCG‐001* among the four groups. (R) The differences in *Ruminococcus gauvreauii group* among the four groups. (S) The differences in *Lachnoclostridium* among the four groups. (T) The differences in *Ruminococcaceae UCG‐010* among the four groups. (U) The differences in *Ruminococcaceae UCG‐013* among the four groups. (V) The differences in *Lachnospiraceae UCG‐008* between the control group and the CUMS group. (W) The differences in *Lachnospiraceae ND3007 group* among the four groups. (X) The differences in *Ruminococcus gnavus CC55 001C* among the four groups. (Y) The differences in *Clostridium* sp. A3LF 105b among the four groups. Statistical analyses were performed using Mann–Whitney *U* test. Violin plots display microbial abundance profiles, with blue shading indicating taxa significantly depleted and green shading highlighting taxa enriched in the CUMS cohort compared to controls. CUMS + AP, the CUMS + acupuncture mice; CUMS, chronic and unpredictable mild stress. *p < 0.05, **p < 0.01, and ***p < 0.001.

Taxonomic analysis revealed that depressive symptoms were predominantly associated with a reduced abundance of *Firmicutes* (Figure 2D,E,H), a phylum displaying an inverse correlation with behavioral deficits. Notably, protective taxa within this phylum included members of the *Lachnospiraceae* family (e.g., *Lachnospiraceae UCG 001*, *UCG 008*, *ND3007 group*, and *[Ruminococcus] gauvreauii group*) (Figure 2Q,R,V,W), the *Ruminococcaceae* family (*Faecalibacterium* and *Ruminococcaceae UCG 002, UCG 009, UCG 010, and UCG 013 clusters*) (Figure 2K,N–P,T,U), and *Christensenellaceae* family (e.g., *Christensenellaceae R7 group*) (Figure 2J,M). Conversely, CUMS mice demonstrated a marked microbial shift characterized by relative expansion of *Bacteroidetes* (principally *Bacteroides* spp.) (Figure 2C,B,G,I), *Lachnoclostridium* (Figure 2S), *Ruminococcus gnavus CC55 001C* (Figure 2X), and *Clostridium* sp. *A3LF 105b* (Figure 2Y) that taxa implicated as potential pathobionts in the CUMS pathology.

Acupuncture intervention partially reversed these dysbiotic alterations, restoring abundances of beneficial *Firmicutes* (Figure 2D) while suppressing CUMS‐associated *Bacteroidia* and *Bacteroidales* (Figure 2A,F). Comparative analysis between groups (Ctrl vs. CUMS; Ctrl + AP vs. CUMS + AP) confirmed that the antidepressive efficacy of acupuncture correlated with microbial community restructuring toward a nondepressed phenotype. These findings suggest that CUMS‐induced behavioral deficits arise in part from a depletion of protective commensals and enrichment of pro‐inflammatory taxa, whereas acupuncture exerts therapeutic effects through microbiota‐dependent rebalancing of these populations.

### 3.3 Acupuncture at the GV20 and ST36 Acupoint Attenuated Depression‐Induced Metabolic Dysregulation

**FIGURE 3:**
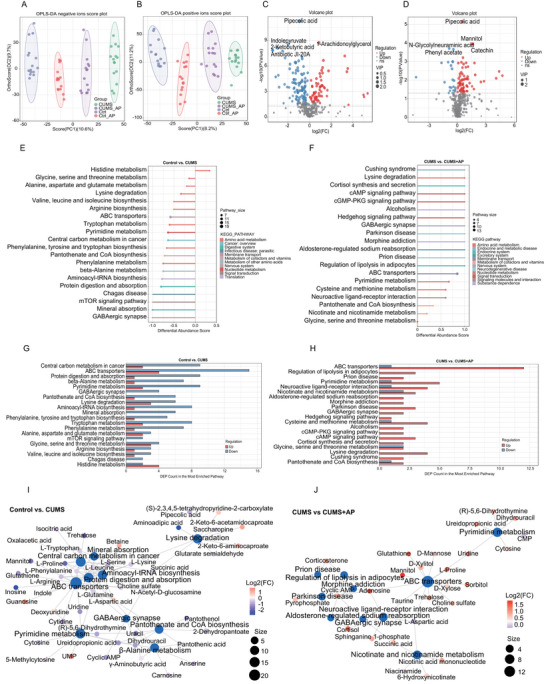
**Acupuncture attenuated depression‐induced gut metabolic disturbances**. (A) The (orthogonal partial least squares discriminant analysis, OPLS‐DA) score plot shown negative ionization mode of the gut metabolites. (B) The OPLS‐DA score plot shown positive ionization mode among the four groups (Bray–Curtis measurements, PERMANOVA). (C) Volcano plot highlighting differentially abundant metabolites between control and CUMS groups (|log2 FC| > 1, *p* < 0.05), with upregulated metabolites (red) and downregulated metabolites (blue). (D) Comparative volcano plot of metabolite shifts between CUMS and CUMS + AP groups (FDR value was calculated by DESeq2). (E and F) KEGG pathway enrichment analyses demonstrating metabolic pathway alterations between control versus CUMS (E) and CUMS versus CUMS + AP (F), quantified through DA scores. (G and H) Distribution of differential metabolites mapped to enriched KEGG pathways for control versus CUMS (G) and CUMS versus CUMS + AP (H). (I and J) Network diagrams visualizing metabolite‐pathway interactions in control versus CUMS (I) and CUMS versus CUMS + AP (J) comparisons. Statistical analyses were performed using Mann–Whitney *U* test. CUMS + AP, the CUMS + acupuncture mice; CUMS, chronic and unpredictable mild stress; FDR, false discovery rate; KEGG, Kyoto Encyclopedia of Genes and Genomes.

Untargeted metabolomics analysis was conducted to assess gut metabolic profiles in mice subjected to CUMS and subsequent acupuncture treatment at GV20 and ST36 acupoints. OPLS‐DA score plots demonstrated distinct clustering patterns, with significant shifts in the gut metabolome of CUMS mice in both positive and negative ion modes (Figure 3A,B). Acupuncture intervention partially restored metabolic profiles toward baseline levels.

Comparative analysis identified 3658 differentially abundant metabolites between CUMS and control mice, including 1157 upregulated and 2501 downregulated species. CUMS mice exhibited marked reductions in four key metabolites: pipecolic acid, indolepyruvate, antibiotic JI‐20A, and 2‐ketobutyric acid, alongside a significant elevation of 1‐arachidonoylglycerol (Figure 3C). Notably, the depletion of neuroprotective metabolites such as pipecolic acid and indolepyruvate may impair systemic resilience to stress and neuroinflammation, exacerbating depressive pathophysiology. In the CUMS + AP group, 2718 differentially regulated metabolites were detected (1640 upregulated and 1078 downregulated). Compared with the CUMS mice alone, two positive ion metabolites pipecolic acid and mannitol were significantly upregulated in the CUMS + AP group. Besides, the plot shows one significantly upregulated negative ion metabolite catechin and two upregulated negative ion metabolite *N*‐glycolylneuraminic acid and phenyl acetate (Figure 3D).

Both KEGG‐enriched DA‐score plots and map of differential metabolite numbers in KEGG pathway revealed that amino acid metabolism (e.g., tryptophan metabolism, phenylalanine metabolism, phenylalanine, tyrosine, and tryptophan biosynthesis) was crucial metabolic pathway that may be a key contributor to depressive behavior (Figure 3E,G). CUMS‐regulated differential metabolites were also embodied GABAergic synapse (Figure 3I). The results indicated that amino acid metabolism disorders and GABAergic synapse may lead to depression‐like behavior in mice. Gut metabolites in CUMS + AP group were identified, which contained neuroactive ligand–receptor interaction, GABAergic synapse, and cortisol synthesis and secretion (Figure 3F). Acupuncture treatment also restored the disturbed amino acid metabolism of depressed mice (Figure 3H). The neuroactive ligand–receptor interaction pathway is a hub connecting antidepressant metabolic pathways (Figure 3J).

**FIGURE 4:**
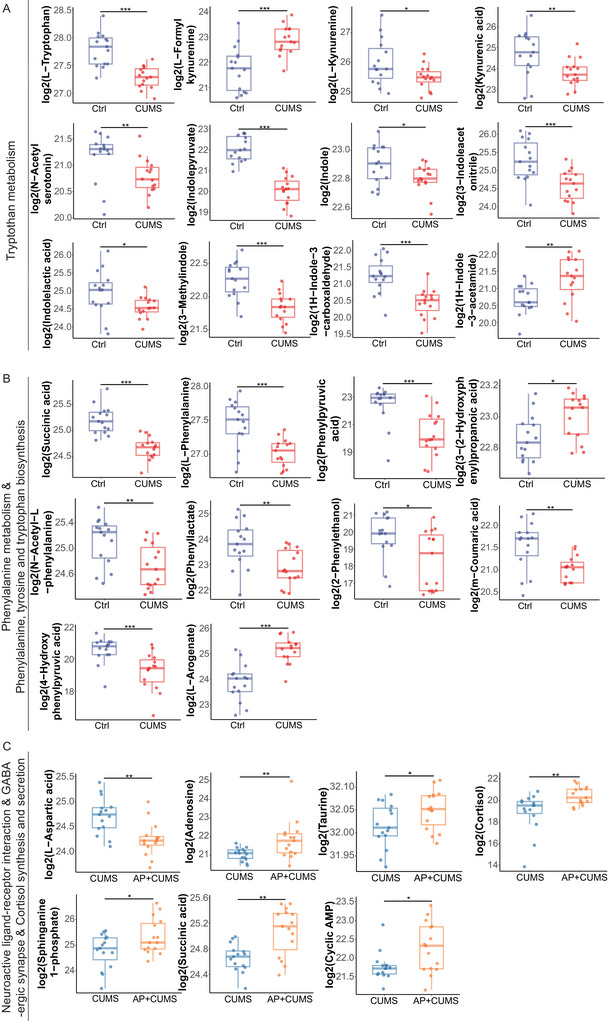
**Pathway‐specific metabolic profiling reveals group‐dependent variations**. (A) Tryptophan pathway metabolites, critical in neuroimmune modulation, encompass l‐tryptophan and its derivatives (l‐formylkynurenine, l‐kynurenine, kynurenic acid, *N*‐acetylserotonin, indolepyruvate, and indole, 3‐indoleacetonitrile). (B) Metabolites of the phenylalanine, tyrosine, and tryptophan biosynthesis and phenylalanine metabolism pathways include l‐phenylalanine, phenylpyruvic acid, l‐arogenate, 4‐hydroxyphenylpyruvic acid, 3‐(2‐hydroxyphenyl) propanoic acid, phenyllactate, *N*‐acetyl‐l‐phenylalanine, 2‐phenylethanol, *m*‐coumaric acid, and succinic acid. (C) Neuroactive ligand–receptor interactions exhibit dysregulation in signaling molecules, including adenosine, taurine, cortisol, and sphinganine 1‐phosphate (S1P). GABAergic synapse‐associated metabolites succinic acid and cyclic AMP display distinct abundance patterns between groups. Statistical analyses were performed using Wilcoxon rank sum test. CUMS + AP, the CUMS + acupuncture mice; CUMS, chronic and unpredictable mild stress. *p < 0.05, **p < 0.01, and ***p < 0.001.

Our metabolomics analysis revealed that tryptophan metabolism is primarily mediated through two distinct pathways: the kynurenine pathway (Figure 4A–D) and the serotonin (5‐hydroxytryptamine, 5‐HT) pathway (Figure 4E,F). Notably, both tryptophan and serotonin levels were significantly reduced in the CUMS group, a phenomenon strongly correlated with the onset of depression‐like behaviors. Concurrently, impairments in the biosynthetic pathways of phenylalanine, tyrosine, and tryptophan (Figure 4A,G,J,K,Q,R) were observed, suggesting broad dysregulation of monoamine neurotransmitter synthesis. These defects may contribute to systemic adverse effects and neuropsychiatric complications. Specifically, tyrosine—the metabolic precursor to dopamine—exhibited pathway disruption, implicating dopamine dysregulation in the observed mood and reward‐processing deficits associated with depression‐like phenotypes. Furthermore, attenuated phenylalanine metabolism (Figure 4I–P) likely exacerbated this cascade by limiting tyrosine availability, thereby compounding depressive symptomology through secondary neurotransmitter imbalances.

Mechanistically, acupuncture at GV20 and ST36 attenuated depression‐like behaviors via neuroactive ligand–receptor interactions (Figure 4S–W). Our data further demonstrate that acupuncture treatment normalized stress‐induced perturbations in cortisol synthesis and secretion (Figure 4V,Y). Intriguingly, succinic acid concentrations within the GABAergic synapse pathway were markedly elevated in acupuncture‐treated depressed mice (Figure 4X,Y). This finding suggests that acupuncture enhances GABAergic synaptic activity—a plausible mechanism underlying its anxiolytic and mood‐stabilizing effects.

### 3.4 Acupuncture Treatment Affects the Relationship Between Intestinal Flora and Metabolites in Depressed Mice

**FIGURE 5:**
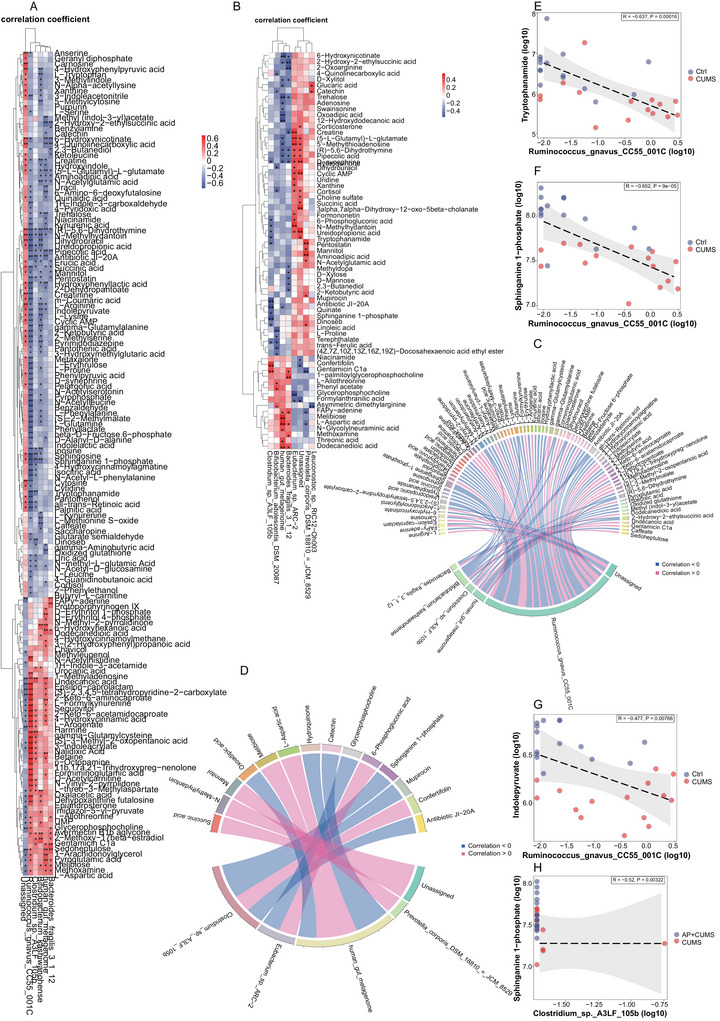
**Acupuncture treatment affects the association between gut flora and metabolites in CUMS mice**. (A and C) Clustered heatmap and circos plot display global microbiota–metabolite correlations in control versus CUMS groups, analyzed by Spearman's rank‐order coefficient. Blue and red represent negative and positive correlations, respectively. Data are represented by the effect size (log fold change). (B and D) Differential correlation (simple spearman analysis, asterisk “*” indicates *p* value <0.05) patterns between CUMS and CUMS + AP groups. AP intervention altered associations, particularly resolving dysregulated interactions in CUMS. (E and G) Scatter plots illustrate distinct correlations of *Ruminococcus gnavus CC55 001C* with tryptophanamide (E), sphinganine 1‐phosphate (F), and 3‐indoleacrylate (G) across control and CUMS cohorts. (H) Scatter plot of correlation between *Clostridium* sp. *A3LF 105b* and sphinganine 1‐phosphate was shown in the CUMS and the CUMS + AP groups. Correlation coefficients were analyzed using Pearson correlation analysis, and least square linear regression lines (black line) with 95% confidence interval (gray shading) are provided for visual representation of the nonparametric testing. CUMS + AP, the CUMS + acupuncture mice; CUMS, chronic and unpredictable mild stress. *p < 0.05, **p < 0.01, and ***p < 0.001.

Spearman correlation analysis of differentially abundant intestinal bacteria and metabolites in CUMS versus control mice demonstrated predominantly negative associations between microbial taxa and metabolic signatures (Figure 5A). Circular network visualization revealed that *R. gnavus CC55 001C* showed the most pronounced upregulation in CUMS mice (Figure 5C), exhibiting strong negative correlations with S1P and tryptophanamide (*p* < 0.001 and *R* < −0.63) (Figure 5E,F), yet positive correlations with 3‐indoleacrylate (*p* < 0.001 and *R* > 0.69) (Figure 5G). These metabolic shifts suggest potential dysregulation of tryptophan metabolism pathways, which are critical for neurotransmitter synthesis and neural homeostasis.

After acupuncture treatment, the intestinal microbiota and metabolites of depressed mice were partially altered (Figure 5B,D). Notably, acupuncture treatment significantly reduced the relative abundance of *Clostridium* sp. *A3LF 105b*, a taxon inversely correlated with S1P levels (*p* < 0.01 and *R* < −0.52) (Figure 5H). Specifically, compared with depressed mice, the abundances of the above pathogenic bacteria and metabolites were significantly reduced after acupuncture treatment, and the abundance of probiotics and beneficial metabolites was significantly increased.

## 4 Discussion

Our study elucidates the therapeutic mechanisms underlying acupuncture at GV20 and ST36 acupoints in ameliorating depressive‐like behaviors in a CUMS mouse model. The CUMS cohort exhibited a characteristic *Bacteroidetes/Firmicutes* ratio elevation, marked by *Bacteroides* and *Lachnoclostridium* enrichment alongside depletion of beneficial taxa, including *Faecalibacterium, Ruminococcaceae UCG 002/009/010/013*, and *Christensenellaceae R7 group, Lachnospiraceae UCG 001/008, Lachnospiraceae ND3007 group*, and *[Ruminococcus] gauvreauii group*. CUMS mice exhibit significantly lower amino acid metabolic pathway compared to the healthy controls. Notably, acupuncture at the acupuncture treatment can activate the neuroactive ligand–receptor interaction, GABAergic synapse, and cortisol synthesis and secretion in the CNS, influencing behavior and modulating neuronal activity.

### 4.1 Gut Microbiome Remodeling

Gut microbiome dysbiosis, particularly involving *Bacteroides* and *Clostridium*, has been implicated in MDD. Elevated *Bacteroides* abundance may exacerbate neuroinflammation through increased peripheral cytokine production and synthesis of the monoaminergic neurotransmitter tryptamine (Hu et al. 2023). *R. gnavus* exhibits strain‐specific effects on host health with its ability to produce SCFAs, tryptophan, and bile acid metabolites, potentially impacting metabolic and neurological disorders (Han et al. 2022). Notably, the mucolytic pathogen *R. gnavus CC55 001C*, positively correlated with depressive severity, demonstrated significant proliferation in CUMS mice (Crost et al. 2023; Yang et al. 2020). This strain's capacity to degrade intestinal mucus barriers and induce pro‐inflammatory cytokine production (IL‐6, TNF‐α) via tryptophan metabolism disruption may facilitate gut‐derived neuroinflammation (Di Luccia et al. 2024; Zhang, Sun, et al. 2024).

In this study, acupuncture effectively ameliorated depressive behaviors and increased *Firmicutes* abundance (Wang, Yang, et al. 2021). Key bacterial families associated with depression include *Christensenellaceae, Lachnospiraceae*, and *Ruminococcaceae*, which mediate the relationship between gut flora imbalance and depression by regulating Th17/Treg cell balance (Humbel et al. 2020; Bosch et al. 2022). *Christensenellaceae R 7 group* was negatively correlated with the Hamilton depression scale 17‐item (HAMD‐17) and Center for Epidemiologic Studies depression scale (CES‐D) scores (Yu et al. 2024). *Faecalibacterium* negatively correlates with depression severity and can reduce inflammatory mediators and enhance short‐chain fatty acid production (Tian et al. 2022). Notably, acupuncture promotes intestinal homeostasis by enriching beneficial taxa such as *Faecalibacterium prausnitzii*, restoring epithelial barrier function, and regulating Th1/Th17 cytokine equilibrium (Simpson et al. 2021). These findings collectively suggest that gut microbiota remodeling serves as a critical mechanism underlying acupuncture's therapeutic potential in depression management.

### 4.2 Neurotransmitter System Modulation

Acupuncture stimulation at acupoints GV20 and ST36 exerts antidepressant effects primarily via neuroactive ligand–receptor interaction pathways (Han et al. 2021). The neuroactive ligand–receptor interaction pathway involves the interactions of a variety of neurotransmitters (e.g., GABA, serotonin, dopamine, and glutamate) and their receptors, which are fundamental to CNS signaling (Wang, Zhou, et al. 2024). Elevated glucocorticoids and diminished cortical GABA may influence stress response and depressive phenotypes in a sex‐dependent manner (Ironside et al. 2024). Vagal ACh‐α7nAChR signaling activates JAK2/STAT3 in intestinal macrophages, reducing inflammation in postoperative ileus (Li et al. 2025). Hepatic vagus nerve signaling promotes liver regeneration via the IL‐6/STAT3 pathway, an effect enhanced by electroacupuncture (Yang et al. 2024). Transcutaneous auricular vagus nerve stimulation (taVNS) suppresses the TLR4/MyD88/NF‐κB pathway in the prefrontal cortex, downregulating pro‐inflammatory cytokines and alleviating inflammation‐induced depression (Zhang, Zhao, et al. 2024; Song et al. 2025). Vagotomy or α7nAChR antagonists (e.g., methyllycaconitine) abolish acupuncture's anti‐inflammatory and prokinetic effects, confirming vagus nerve mediation (Zhang, Zhang, et al. 2022). Acupuncture may have therapeutic effects on depression by recovering the release of neurotransmitters and the activity of receptors.

Acupuncture demonstrates significant efficacy in improving depression symptoms compared to sham acupuncture controls across multiple randomized controlled trials (RCTs), though methodological challenges in designing inert sham controls must be acknowledged. Future research on the placebo effects can adopt the following approaches. Prioritize non‐penetrating sham devices applied to clearly defined non‐acupoints to minimize physiological activity (Xia et al. 2025). Rigorously assess and report blinding success (e.g., using Bang blinding index) (Yin et al. 2022). Include both a sham control and a no‐treatment or standard care‐only control arm to help differentiate specific effects from nonspecific effects and natural history (Cohen et al. 2024). Measure and account for patient expectations regarding treatment, as high expectancy can influence outcomes in both real and sham groups (Zhang et al. 2023). Incorporate neuroimaging (e.g., fMRI) or biomarker assessments to objectively demonstrate physiological changes associated with real acupuncture beyond placebo responses (Wu et al. 2024).

Acupuncture elevates S1P levels by restoring gut microbiota (e.g., *F. prausnitzii* and *Roseburia faecis*), which in turn strengthens intestinal barrier function and reduces systemic inflammation (Luo et al. 2025). Conversely, SSRIs disrupt microbial ecology, promote antibiotic resistance gene transfer, and exacerbate gut dysbiosis (Ding et al. 2025). S1P, bound to apolipoprotein M (ApoM) on HDL, activates anti‐inflammatory pathways (e.g., SIRT‐1 and STAT‐3) in the brain, suppresses neuroinflammation, and shifts microglia from pro‐inflammatory M1 to anti‐inflammatory M2 phenotypes, thereby inhibiting NLRP3 inflammasome assembly and IL‐1β/TNF‐α release (Morris et al. 2021; Guo et al. 2020). Fingolimod, an S1P receptor (S1PR) modulator, replicates this effects, confirming the role of S1P in stress resilience (Wang, Bi, et al. 2024). SSRIs lack this specific immunomodulatory action. Furthermore, acupuncture elevates hippocampal S1P levels in depressed rats, activating S1PR1 and downstream BDNF‐TrkB signaling, which promotes synaptic plasticity and neurogenesis (Luo et al. 2025). S1P also enhances BDNF expression and cognitive function, highlighting broader neurorestorative effects absent in SSRIs.

The S1P‐microbiota axis offers several translational advantages. Unlike SSRIs, acupuncture's S1P‐driven pathway enhances neuroprotection without antimicrobial resistance (AMR) while preserving microbiome health (Cong et al. 2025). SSRIs induce dysbiosis, increasing *Enterobacteriaceae* and reducing anti‐inflammatory butyrate producers (Simpson et al. 2021). Acupuncture restores microbial and metabolic homeostasis. Acupuncture concurrently repairs gut barrier integrity (reducing LPS/DAO), enriches SCFA‐producing bacteria, and suppresses Th1/Th17 inflammation (Bao et al. 2022; Li et al. 2024). Due to SSRIs’ gastrointestinal/CNS adverse events, acupuncture is supported by long‐term using in comorbid gastrointestinal symptoms in depression (e.g., IBD‐associated depression) (Cui et al. 2024). Plasma S1P levels could serve as a biomarker for treatment response, personalizing acupuncture protocols for refractory depression (Morris et al. 2021). In summary, acupuncture operates through a microbiota–S1P–immune mechanism that concurrently targets gut–brain communication, neuroinflammation, and neural plasticity. This represents a paradigm shift from monoamine‐based strategies and offers a promising alternative for treatment‐resistant depression with favorable safety and multisystem efficacy.

### 4.3 Study Limitations and Future Directions

The combination of 16sRNA sequencing and metabolic analysis is a proven strategy for revealing the taxonomic and functional characteristics of the gut microbiome. Additionally, analyses typically rely on stool samples, preventing correlation with gut‐specific ecological/nutritional niche strains. The efficacy of using acupuncture to treat depression has only been validated in animal studies, not clinical trials. Another limitation is the lack of external validation data sets. Most correlations are based on 16S rRNA amplicon sequencing of fecal microbiota, revealing bacterial relative abundance at various taxonomic levels but lacking species or strain resolution. We realize that our findings do not prove causation, although we are primarily focused on biologically plausible mechanisms. These findings suggest that the acupuncture is efficient for mouse with depression, but additional studies with larger samples and longer interventions are needed for confirmation. Although this study focused on male mice to mitigate hormonal confounders, future work will incorporate synchronized estrous cycle staging in female cohorts to elucidate sex‐specific mechanisms. Longitudinal assessments across ovarian hormone phases are critical to disentangle organizational and activational effects of acupuncture on gut–brain communication.

## 5 Conclusion

This work establishes a gut–microbiome–brain axis through which acupuncture alleviates depression pathophysiology. By simultaneously targeting normalization of gut microbial dysbiosis, restoration of amino acid metabolic homeostasis, and modulation of neuroactive ligand–receptor interactions, this ancient modality demonstrates multidimensional therapeutic potential worthy of further clinical exploration.

## Author Contributions

Ruina Liu and Hao Zhu conceived and designed the project. Jiarong Tian, Cailing Wei, and Yijun Li participated in animal experiments. Pu Lei, Yuanyuan Ding, and Wen Lu managed the literature searches and analyses. Cailing Wei and Xiaoyan He performed the data analyses. Cailing Wei interpreted the results and wrote the manuscript, which was revised by Ruina Liu and Hao Zhu. All authors contributed to the revision of the article. Ya'ni Yang provided financial support and offered valuable suggestions for the revision of the article.

## Funding

This research was partially sponsored by the National Natural Science Foundation of China, including the General Program (No. 82271572), the Excellent Young Science Fund (No. 82022023), the Young Science Fund (No. 82401774), and the Basic Research of Natural Science Fund of Shaanxi Province (No. S2024‐JC‐QN‐2742), as well as the Xi'an Science and Technology Program (22YXYJ0023). The funding sources had the role in study design; in the collection, analysis, and interpretation of data; in the report's writing; and in the decision to submit the article for publication.

## Ethics Statement

All animal experiment procedures have been approved by the Experimental Animal Ethics Committee of Xi'an Jiaotong University (No. 2022‐497).

## Conflicts of Interest

The authors declare no conflicts of interest.

## Peer Review

The peer review history for this article is available at https://publons.com/publon/10.1002/brb3.71022.

## Data Availability

The data that support the findings of this study are available on request from the corresponding author. NCBI accession numbers for 16S data is PRJNA1280274.

## References

[brb371022-bib-0001] Abdelrazig, S. , L. Safo , G. A. Rance , et al. 2020. “Metabolic Characterisation of Magnetospirillum Gryphiswaldense MSR‐1 Using LC–MS‐Based Metabolite Profiling.” RSC Advances 10, no. 54: 32548–32560. 10.1039/d0ra05326k. 35516490 PMC9056635

[brb371022-bib-0002] Bao, C. , L. Wu , D. Wang , et al. 2022. “Acupuncture Improves the Symptoms, Intestinal Microbiota, and Inflammation of Patients With Mild to Moderate Crohn's Disease: A Randomized Controlled Trial.” Eclinicalmedicine 45: 101300. 10.1016/j.eclinm.2022.101300. 35198926 PMC8850329

[brb371022-bib-0003] Bosch, J. A. , M. Nieuwdorp , A. H. Zwinderman , et al. 2022. “The Gut Microbiota and Depressive Symptoms Across Ethnic Groups.” Nature Communications 13, no. 1: 7129. 10.1038/s41467-022-34504-1. PMC9726934 36473853

[brb371022-bib-0004] Chang, H. , M. Huo , Q. Zhang , et al. 2023. “Flexible Needle‐Type Microbiosensor for Real‐Time Monitoring Traditional Acupuncture‐Mediated Adenosine Release In Vivo.” Biosensors and Bioelectronics 235: 115383. 10.1016/j.bios.2023.115383. 37207583

[brb371022-bib-0005] Chen, K. B. , Y. Huang , X. L. Jin , and G. F. Chen . 2019. “Electroacupuncture or Transcutaneous Electroacupuncture for Postoperative Ileus After Abdominal Surgery: A Systematic Review and Meta‐Analysis.” International Journal of Surgery 70: 93–101. 10.1016/j.ijsu.2019.08.034. 31494334

[brb371022-bib-0006] Chen, L. , Z. Liu , Z. Zhao , et al. 2023. “Dopamine Receptor 1 on CaMKII‐Positive Neurons Within Claustrum Mediates Adolescent Cocaine Exposure‐Induced Anxiety‐Like Behaviors and Electro‐Acupuncture Therapy.” Theranostics 13, no. 10: 3149–3164. 10.7150/thno.83079. 37351159 PMC10283049

[brb371022-bib-0007] Cohen, L. , S. C. Danhauer , M. K. Garcia , et al. 2024. “Acupuncture for Chronic Radiation‐Induced Xerostomia in Head and Neck Cancer.” JAMA Network Open 7, no. 5: e2410421. 10.1001/jamanetworkopen.2024.10421. 38739392 PMC11091764

[brb371022-bib-0008] Cong, L. , S. Ding , Y. Guo , et al. 2025. “Acupuncture Alleviates CSDS‐Induced Depressive‐Like Behaviors by Modulating Synaptic Plasticity in vCA1.” Theranostics 15, no. 10: 4808–4822. 10.7150/thno.106751. 40225589 PMC11984413

[brb371022-bib-0009] Crost, E. H. , E. Coletto , A. Bell , and N. Juge . 2023. “Ruminococcus Gnavus: Friend or Foe for Human Health.” FEMS Microbiology Reviews 47, no. 2: fuad014. 10.1093/femsre/fuad014. 37015876 PMC10112845

[brb371022-bib-0010] Cui, L. , S. Li , S. Wang , et al. 2024. “Major Depressive Disorder: Hypothesis, Mechanism, Prevention and Treatment.” Signal Transduction and Targeted Therapy 9, no. 1: 30. 10.1038/s41392-024-01738-y. 38331979 PMC10853571

[brb371022-bib-0011] De Oliveira Rodrigues, D. M. , P. R. Menezes , A. E. Machado Ribeiro Silotto , et al. 2023. “Efficacy and Safety of Auricular Acupuncture for Depression: A Randomized Clinical Trial.” JAMA Network Open 6, no. 11: e2345138. 10.1001/jamanetworkopen.2023.45138. 38032640 PMC10690462

[brb371022-bib-0012] Di Luccia, B. , M. Molgora , D. Khantakova , et al. 2024. “TREM2 Deficiency Reprograms Intestinal Macrophages and Microbiota to Enhance Anti‐PD‐1 Tumor Immunotherapy.” Science Immunology 9, no. 95: eadi5374. 10.1126/sciimmunol.adi5374. 38758808 PMC11299520

[brb371022-bib-0013] Ding, P. , J. Lu , T. Lei , et al. 2025. “Antidepressant Drugs Promote the Spread of Broad‐Host‐Range Plasmid in Mouse and Human Gut Microbiota.” Gut Microbes 17, no. 1: 2514138. 10.1080/19490976.2025.2514138. 40462285 PMC12143698

[brb371022-bib-0014] Gagnebin, Y. , D. Tonoli , P. Lescuyer , et al. 2017. “Metabolomic Analysis of Urine Samples by UHPLC‐QTOF‐MS: Impact of Normalization Strategies.” Analytica Chimica Acta 955: 27–35. 10.1016/j.aca.2016.12.029. 28088278

[brb371022-bib-0015] Guo, Y. , X. Gan , H. Zhou , et al. 2020. “Fingolimod Suppressed the Chronic Unpredictable Mild Stress‐Induced Depressive‐Like Behaviors via Affecting Microglial and NLRP3 Inflammasome Activation.” Life Sciences 263: 118582. 10.1016/j.lfs.2020.118582. 33058911

[brb371022-bib-0016] Han, L. , L. Zhao , Y. Zhou , et al. 2022. “Altered Metabolome and Microbiome Features Provide Clues in Understanding Irritable Bowel Syndrome and Depression Comorbidity.” ISME Journal 16, no. 4: 983–996. 10.1038/s41396-021-01123-5. 34750528 PMC8940891

[brb371022-bib-0017] Han, Z. , Y. Zhang , P. Wang , Q. Tang , and K. Zhang . 2021. “Is Acupuncture Effective in the Treatment of COVID‐19 Related Symptoms? Based on Bioinformatics/Network Topology Strategy.” Briefings in Bioinformatics 22, no. 5: bbab110. 10.1093/bib/bbab110. 33866350 PMC8083275

[brb371022-bib-0018] Hao, W. , Q. Ma , L. Wang , et al. 2024. “Gut Dysbiosis Induces the Development of Depression‐Like Behavior Through Abnormal Synapse Pruning in Microglia‐Mediated by Complement C3.” Microbiome 12, no. 1: 34. 10.1186/s40168-024-01756-6. 38378622 PMC10877840

[brb371022-bib-0019] Horai, H. , M. Arita , S. Kanaya , et al. 2010. “MassBank: A Public Repository for Sharing Mass Spectral Data for Life Sciences.” Journal of Mass Spectrometry 45, no. 7: 703–714. 10.1002/jms.1777. 20623627

[brb371022-bib-0020] Hu, X. , X. Sun , Y. Zhao , et al. 2023. “GlcNac Produced by the Gut Microbiome Enhances Host Influenza Resistance by Modulating NK Cells.” Gut Microbes 15, no. 2: 2271620. 10.1080/19490976.2023.2271620. 37953509 PMC10730189

[brb371022-bib-0021] Humbel, F. , J. H. Rieder , Y. Franc , et al. 2020. “Association of Alterations in Intestinal Microbiota With Impaired Psychological Function in Patients With Inflammatory Bowel Diseases in Remission.” Clinical Gastroenterology and Hepatology 18, no. 9: 2019–2029.e11. 10.1016/j.cgh.2019.09.022. 31546058

[brb371022-bib-0022] Ironside, M. , J. M. Duda , A. D. Moser , et al. 2024. “Association of Lower Rostral Anterior Cingulate GABA+ and Dysregulated Cortisol Stress Response With Altered Functional Connectivity in Young Adults With Lifetime Depression: A Multimodal Imaging Investigation of Trait and State Effects.” American Journal of Psychiatry 181, no. 7: 639–650. 10.1176/appi.ajp.20230382. 38685857 PMC11216878

[brb371022-bib-0023] Jiang, X. , X. Wang , M. Zhang , et al. 2024. “Associations Between Specific Dietary Patterns, Gut Microbiome Composition, and Incident Subthreshold Depression in Chinese Young Adults.” Journal of Advanced Research 65: 183–195. 10.1016/j.jare.2024.05.030. 38879123 PMC11518947

[brb371022-bib-0024] Li, P. , J. Zhao , X. Wei , et al. 2024. “Acupuncture May Play a Key Role in Anti‐Depression Through Various Mechanisms in Depression.” Chinese Medicine 19, no. 1: 135. 10.1186/s13020-024-00990-2. 39367470 PMC11451062

[brb371022-bib-0025] Li, S. , N. Zou , B. Feng , et al. 2025. “Transcutaneous Auricular Vagus Nerve Stimulation Improves Gastric Motility and Visceral Hypersensitivity in Rodents of Functional Dyspepsia by Balancing Duodenal Immune Response: An Experimental Study.” International Journal of Surgery 111, no. 1: 1517–1520. 10.1097/js9.0000000000001984. 39093860 PMC11745774

[brb371022-bib-0026] Liu, P. , Z. Liu , J. Wang , et al. 2024. “Immunoregulatory Role of the Gut Microbiota in Inflammatory Depression.” Nature Communications 15, no. 1: 3003. 10.1038/s41467-024-47273-w. PMC11001948 38589368

[brb371022-bib-0027] Liu, S. , Z. Wang , Y. Su , et al. 2021. “A Neuroanatomical Basis for Electroacupuncture to Drive the Vagal–Adrenal Axis.” Nature 598, no. 7882: 641–645. 10.1038/s41586-021-04001-4. 34646018 PMC9178665

[brb371022-bib-0028] Lu, P. , R. He , Y. Wu , et al. 2025. “Urinary Metabolic Alterations Associated With Occupational Exposure to Metals and Polycyclic Aromatic Hydrocarbons Based on Non‐target Metabolomics.” Journal of Hazardous Materials 487: 137158. 10.1016/j.jhazmat.2025.137158. 39798303

[brb371022-bib-0029] Luo, X.‐Y. , M. Yu , H.‐J. Li , X.‐Y. Kong , Z.‐M. Zou , and X.‐C. Ye . 2025. “Structural Characteristics and Potential Antidepressant Mechanism of a Water‐Insoluble β‐1,3‐Glucan From an Edible Fungus *Wolfiporia cocos* .” Carbohydrate Polymers 348: 122779. 10.1016/j.carbpol.2024.122779. 39562060

[brb371022-bib-0030] Morris, G. , B. K. Puri , C. C. Bortolasci , et al. 2021. “The Role of High‐Density Lipoprotein Cholesterol, Apolipoprotein A and Paraoxonase‐1 in the Pathophysiology of Neuroprogressive Disorders.” Neuroscience & Biobehavioral Reviews 125: 244–263. 10.1016/j.neubiorev.2021.02.037. 33657433

[brb371022-bib-0031] Mu, X. , L. Feng , Q. Wang , et al. 2025. “Decreased Gut Microbiome‐Derived Indole‐3‐Propionic Acid Mediates the Exacerbation of Myocardial Ischemia/Reperfusion Injury Following Depression via the Brain‐Gut‐Heart Axis.” Redox Biology 81: 103580. 10.1016/j.redox.2025.103580. 40058066 PMC11930714

[brb371022-bib-0032] Navarro‐Reig, M. , J. Jaumot , A. García‐Reiriz , and R. Tauler . 2015. “Evaluation of Changes Induced in Rice Metabolome by Cd and Cu Exposure Using LC–MS With XCMS and MCR‐ALS Data Analysis Strategies.” Analytical and Bioanalytical Chemistry 407, no. 29: 8835–8847. 10.1007/s00216-015-9042-2. 26403240

[brb371022-bib-0033] Ogata, H. , S. Goto , K. Sato , W. Fujibuchi , H. Bono , and M. K. Kanehisa . 1999. “Kyoto Encyclopedia of Genes and Genomes.” Nucleic Acids Research 27, no. 1: 29–34. 10.1093/nar/27.1.29. 9847135 PMC148090

[brb371022-bib-0034] Qin, Z. , K. Xiang , D. F. Su , Y. Sun , and X. Liu . 2020. “Activation of the Cholinergic Anti‐Inflammatory Pathway as a Novel Therapeutic Strategy for COVID‐19.” Frontiers in Immunology 11: 595342. 10.3389/fimmu.2020.595342. 33633726 PMC7901247

[brb371022-bib-0035] Simpson, C. A. , C. Diaz‐Arteche , D. Eliby , O. S. Schwartz , J. G. Simmons , and C. S. M. Cowan . 2021. “The Gut Microbiota in Anxiety and Depression—A Systematic Review.” Clinical Psychology Review 83: 101943. 10.1016/j.cpr.2020.101943. 33271426

[brb371022-bib-0036] Smith, C. A. , E. J. Want , G. O'Maille , R. Abagyan , and G. Siuzdak . 2006. “XCMS: Processing Mass Spectrometry Data for Metabolite Profiling Using Nonlinear Peak Alignment, Matching, and Identification.” Analytical Chemistry 78, no. 3: 779–787. 10.1021/ac051437y. 16448051

[brb371022-bib-0037] Song, X. , H. Zhu , Z. Chen , et al. 2025. “Transcutaneous Auricular Vagus Nerve Stimulation Alleviates Inflammation‐Induced Depression by Modulating Peripheral‐Central Inflammatory Cytokines and the NF‐κB Pathway in Rats.” Frontiers in Immunology 16: 1536056. 10.3389/fimmu.2025.1536056. 40453075 PMC12122300

[brb371022-bib-0038] Sud, M. , E. Fahy , D. Cotter , et al. 2007. “LMSD: LIPID MAPS Structure Database.” Nucleic Acids Research 35, no. Database Issue: D527–D532. 10.1093/nar/gkl838. 17098933 PMC1669719

[brb371022-bib-0039] Sun, H. , K. Sun , H. Tian , et al. 2024. “Integrated Metagenomic and Metabolomic Analysis Reveals Distinctive Stage‐Specific Gut‐Microbiome‐Derived Metabolites in Intracranial Aneurysms.” Gut 73, no. 10: 1662–1674. 10.1136/gutjnl-2024-332245. 38960582

[brb371022-bib-0040] Tian, T. , Q. Mao , J. Xie , et al. 2022. “Multi‐Omics Data Reveals the Disturbance of Glycerophospholipid Metabolism Caused by Disordered Gut Microbiota in Depressed Mice.” Journal of Advanced Research 39: 135–145. 10.1016/j.jare.2021.10.002. 35777903 PMC9263645

[brb371022-bib-0041] Wang, H. L. , F. L. Liu , R. Q. Li , et al. 2021. “Electroacupuncture Improves Learning and Memory Functions in a Rat Cerebral Ischemia/Reperfusion Injury Model Through PI3K/Akt Signaling Pathway Activation.” Neural Regeneration Research 16, no. 6: 1011–1016. 10.4103/1673-5374.300454. 33269744 PMC8224106

[brb371022-bib-0042] Wang, J.‐M. , M.‐X. Yang , Q.‐F. Wu , et al. 2021. “Improvement of Intestinal Flora: Accompany With the Antihypertensive Effect of Electroacupuncture on Stage 1 Hypertension.” Chinese Medicine 16, no. 1: 7. 10.1186/s13020-020-00417-8. 33413552 PMC7792359

[brb371022-bib-0043] Wang, L. , E. Hou , L. Wang , et al. 2015. “Reconstruction and Analysis of Correlation Networks Based on GC–MS Metabolomics Data for Young Hypertensive Men.” Analytica Chimica Acta 854: 95–105. 10.1016/j.aca.2014.11.009. 25479872 PMC4432937

[brb371022-bib-0044] Wang, L. , L. Bi , Y. Qiu , et al. 2024. “Effectiveness of Electro‐Acupuncture for Cognitive Improvement on Alzheimer's Disease Quantified via PET Imaging of Sphingosine‐1‐Phosphate Receptor 1.” Alzheimer's & Dementia 20, no. 12: 8331–8345. 10.1002/alz.14260. PMC11667549 39320044

[brb371022-bib-0045] Wang, X. , J. Zhou , T. Jiang , and J. Xu . 2024. “Deciphering the Therapeutic Potential of SheXiangXinTongNing: Interplay Between Gut Microbiota and Brain Metabolomics in a CUMS Mice Model, With a Focus on Tryptophan Metabolism.” Phytomedicine 129: 155584. 10.1016/j.phymed.2024.155584. 38704913

[brb371022-bib-0046] Wishart, D. S. , D. Tzur , C. Knox , et al. 2007. “HMDB: the Human Metabolome Database.” Nucleic Acids Research 35, no. Database Issue: D521–D526. 10.1093/nar/gkl923. 17202168 PMC1899095

[brb371022-bib-0047] Wu, X. , M. Tu , Z. Yu , et al. 2024. “The Efficacy and Cerebral Mechanism of Intradermal Acupuncture for Major Depressive Disorder: A Multicenter Randomized Controlled Trial.” Neuropsychopharmacology 50, no. 7: 1075–1083. 10.1038/s41386-024-02036-5. 39648209 PMC12089605

[brb371022-bib-0048] Xia, J. , and D. S. Wishart . 2011. “Web‐Based Inference of Biological Patterns, Functions and Pathways From Metabolomic Data Using MetaboAnalyst.” Nature Protocols 6, no. 6: 743–760. 10.1038/nprot.2011.319. 21637195

[brb371022-bib-0049] Xia, R. , K. Linde , T. Freilinger , et al. 2025. “Acupuncture for the Prevention of Episodic Migraine.” Cochrane Database of Systematic Reviews 2025, no. 2: CD001218. 10.1002/14651858.Cd015528. PMC11812084 39932102

[brb371022-bib-0050] Xue, N. Y. , D. Y. Ge , R. J. Dong , H. H. Kim , X. J. Ren , and Y. Tu . 2021. “Effect of Electroacupuncture on Glial Fibrillary Acidic Protein and Nerve Growth Factor in the Hippocampus of Rats With Hyperlipidemia and Middle Cerebral Artery Thrombus.” Neural Regeneration Research 16, no. 1: 137–142. 10.4103/1673-5374.286973. 32788468 PMC7818884

[brb371022-bib-0051] Yang, J. , P. Zheng , Y. Li , et al. 2020. “Landscapes of Bacterial and Metabolic Signatures and Their Interaction in Major Depressive Disorders.” Science Advances 6, no. 49: eaba8555. 10.1126/sciadv.aba8555. 33268363 PMC7710361

[brb371022-bib-0052] Yang, L. , Y. Zhou , Z. Huang , et al. 2024. “Electroacupuncture Promotes Liver Regeneration by Activating DMV Acetylcholinergic Neurons‐Vagus‐Macrophage Axis in 70% Partial Hepatectomy of Mice.” Advanced Science (Weinh) 11: e2402856. 10.1002/advs.202402856. PMC11348175 38923873

[brb371022-bib-0053] Yin, X. , W. Li , T. Liang , et al. 2022. “Effect of Electroacupuncture on Insomnia in Patients With Depression: A Randomized Clinical Trial.” JAMA Network Open 5, no. 7: e2220563. 10.1001/jamanetworkopen.2022.20563. 35797047 PMC9264041

[brb371022-bib-0054] Yu, Z. , J. Xian , L. Wang , and H. Yu . 2024. “Effect of Umbilical Moxibustion on Intestinal Flora in Patients With Subthreshold Depression.” Zhongguo Zhenjiuxue 44, no. 8: 881–888. 10.13703/j.0255-2930.20231019-0003. 39111785

[brb371022-bib-0055] Zhang, J. , Z. Qin , T. H. So , et al. 2023. “Acupuncture for Chemotherapy‐Associated Insomnia in Breast Cancer Patients: An Assessor‐Participant Blinded, Randomized, Sham‐Controlled Trial.” Breast Cancer Research 25, no. 1: 49. 10.1186/s13058-023-01645-0. 37101228 PMC10134666

[brb371022-bib-0056] Zhang, N. , Y. Shen , W. Zhu , et al. 2022. “Spatial Transcriptomics Shows Moxibustion Promotes Hippocampus Astrocyte and Neuron Interaction.” Life Sciences 310: 121052. 10.1016/j.lfs.2022.121052. 36220370

[brb371022-bib-0057] Zhang, S. , Y. Sun , Q. Nie , et al. 2024. “Effects of Four Food Hydrocolloids on Colitis and Their Regulatory Effect on Gut Microbiota.” Carbohydrate Polymers 323: 121368. 10.1016/j.carbpol.2023.121368. 37940266

[brb371022-bib-0058] Zhang, S. , Y. Zhao , Z. Qin , et al. 2024. “Transcutaneous Auricular Vagus Nerve Stimulation for Chronic Insomnia Disorder.” JAMA Network Open 7, no. 12: e2451217. 10.1001/jamanetworkopen.2024.51217. 39680406 PMC11650411

[brb371022-bib-0059] Zhang, Y. , Q. Fan , Y. Hou , et al. 2022. “Bacteroides Species Differentially Modulate Depression‐Like Behavior via Gut‐Brain Metabolic Signaling.” Brain, Behavior, and Immunity 102: 11–22. 10.1016/j.bbi.2022.02.007. 35143877

[brb371022-bib-0060] Zhang, Y. , R. Huang , M. Cheng , et al. 2019. “Gut Microbiota From NLRP3‐Deficient Mice Ameliorates Depressive‐Like Behaviors by Regulating Astrocyte Dysfunction via circHIPK2.” Microbiome 7, no. 1: 116. 10.1186/s40168-019-0733-3. 31439031 PMC6706943

[brb371022-bib-0061] Zhang, Z. J. , S. Y. Zhang , X. J. Yang , et al. 2022. “Transcutaneous Electrical Cranial‐Auricular Acupoint Stimulation Versus Escitalopram for Mild‐to‐Moderate Depression: An Assessor‐Blinded, Randomized, Non‐Inferiority Trial.” Psychiatry and Clinical Neurosciences 77, no. 3: 168–177. 10.1111/pcn.13512. 36445151

